# Supplementary material for: Optimized Protocol for Isolation of Small Extracellular Vesicles from Human and Murine Lymphoid Tissues
Source: Int J Mol Sci. 2020 Aug 4;21(15):5586. doi: 10.3390/ijms21155586 (PMC7432511; doi:10.3390/ijms21155586)
Supplement: Supplementary file 1 [file ijms-21-05586-s001.pdf]

Supplemental Data –Optimized protocol for isolation of exosomes from human and murine lymphoid tissues. Bordas et al. IJMS 2020.

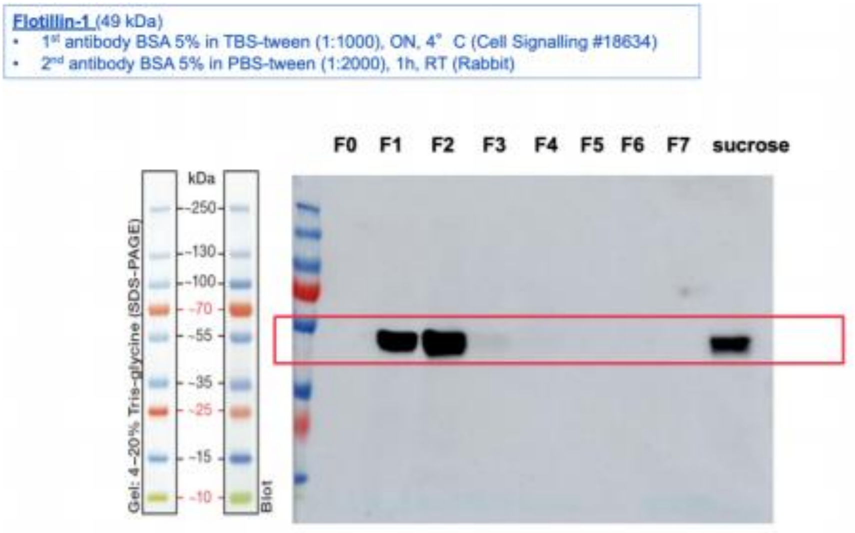

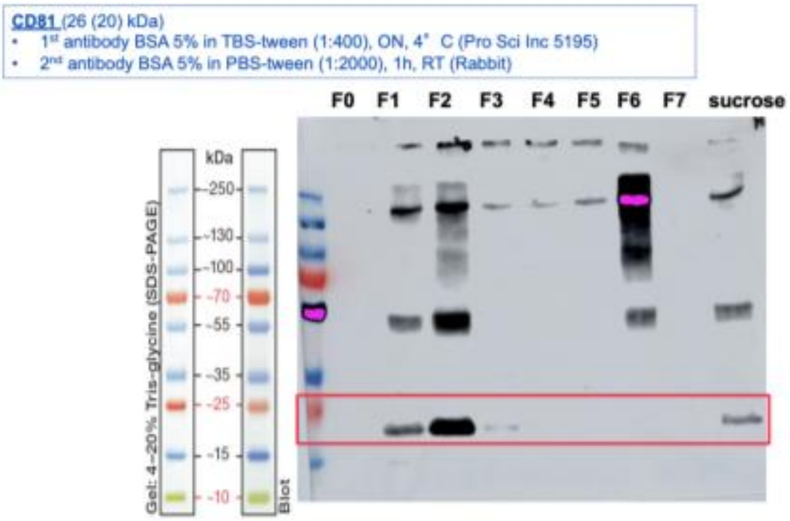

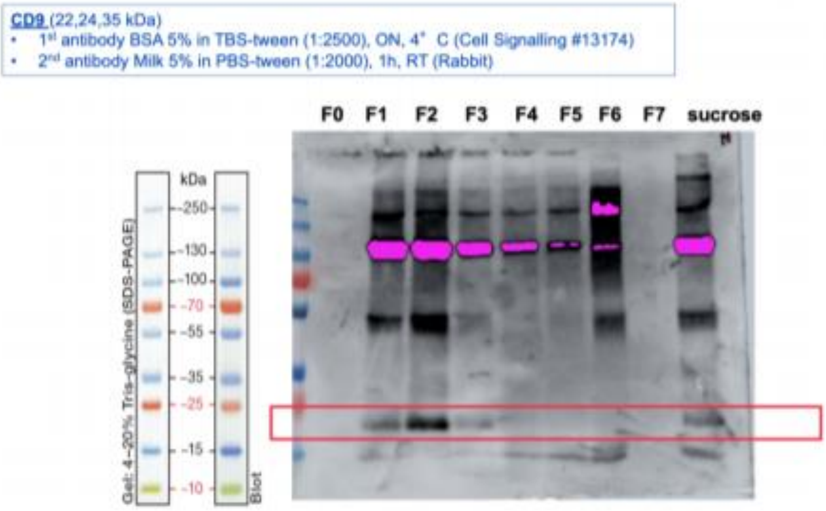

GM130(140 kDa – predicted 112 kDa)
- 1st antibody BSA 5% in TBS-tween (1:1000), ON, 4° C (Cell Signalling # 12480)
- 2nd antibody Milk 5% in PBS-tween (1:2000), 1h, RT (Rabbit)

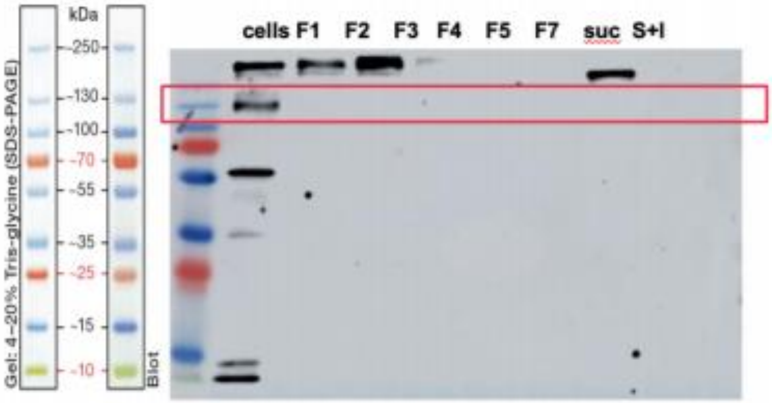

Cytochrome c (114 kDa)
- 1st antibody BSA 5% in TBS-tween (1:1000), ON, 4° C (Cell Signalling #)
- 2nd antibody Milk 5% in PBS-tween (1:2000), 1h, RT (Rabbit)

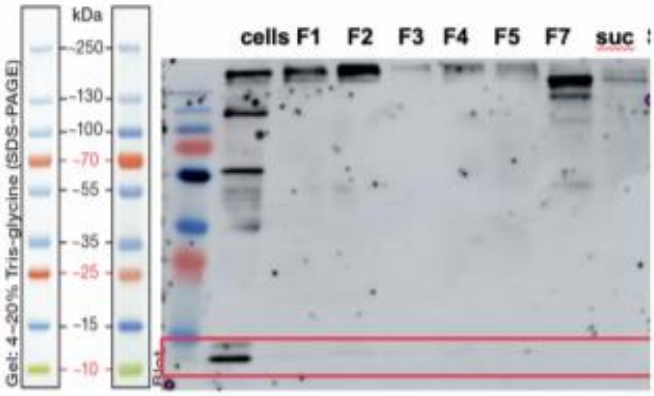

Calnexin (90 kDa)
- 1st antibody BSA 5% in TBS-tween (1:500), ON, 4° C (Gene Script, A-01240-40)
- 2nd antibody BSA 5% in PBS-tween (1:2000), 1h, RT (Rabbit)

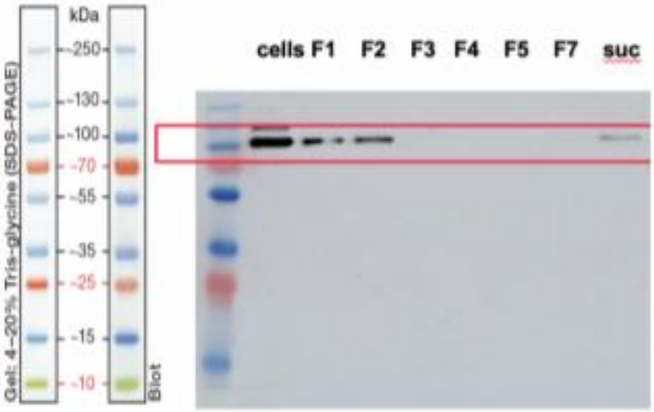

Figure S1. Immunoblot from human Lymph Nodes.

CD81 (26 (20) kDa)
- 1st antibody BSA 5% in TBS-tween (1:400), ON, 4° C (Pro Sci Inc 5195)
- 2nd antibody BSA 5% in PBS-tween (1:2000), 1h, RT (Rabbit)

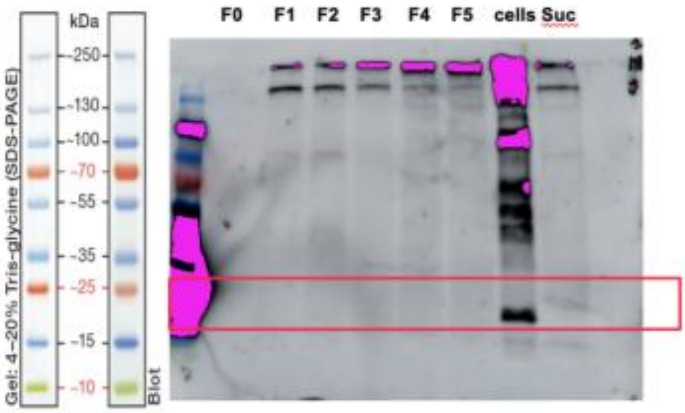

Alix (95 kDa)
- 1st antibody BSA 5% in TBS-tween (1:1000), ON, 4° C (Cell Signalling #2171)
- 2nd antibody BSA 5% in PBS-tween (1:2000), 1h, RT (Mouse)

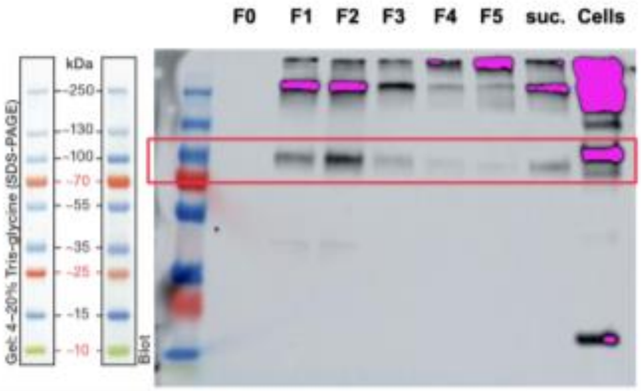

ATPa5 (44 kDa)
- 1st antibody BSA 5% in TBS-tween (1:1000), ON, 4° C (Abcam)
- 2nd antibody Milk 5% in PBS-tween (1:2000), 1h, RT (Mouse)

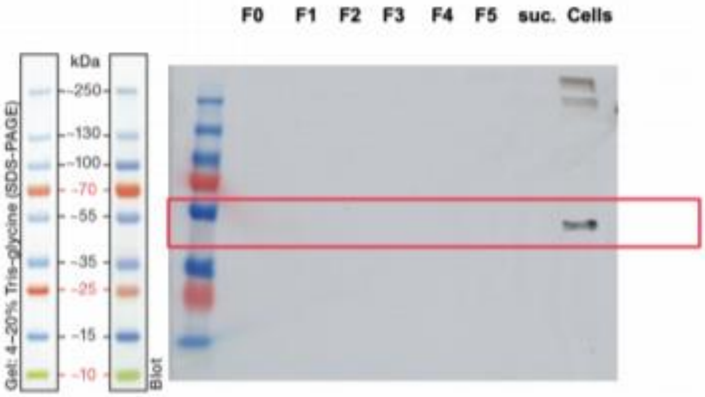

Flotillin-1 (49 kDa)
- 1st antibody BSA 5% in TBS-tween (1:1000), ON, 4° C (Cell Signalling #18634)
- 2nd antibody BSA 5% in PBS-tween (1:2000), 1h, RT (Rabbit)

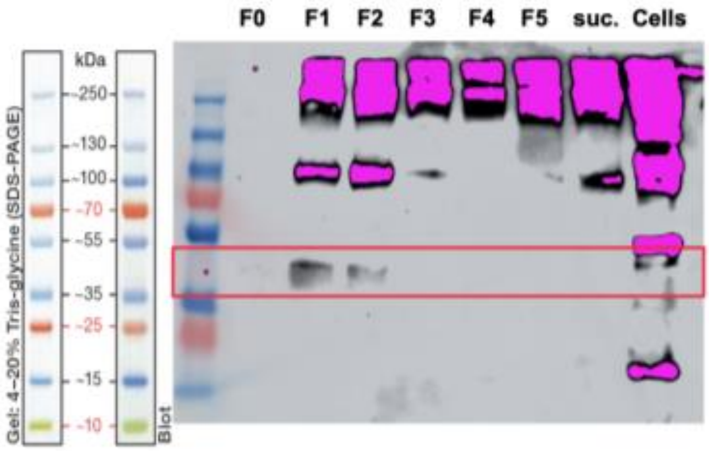

Figure S2. Immunoblot from murine Spleens.

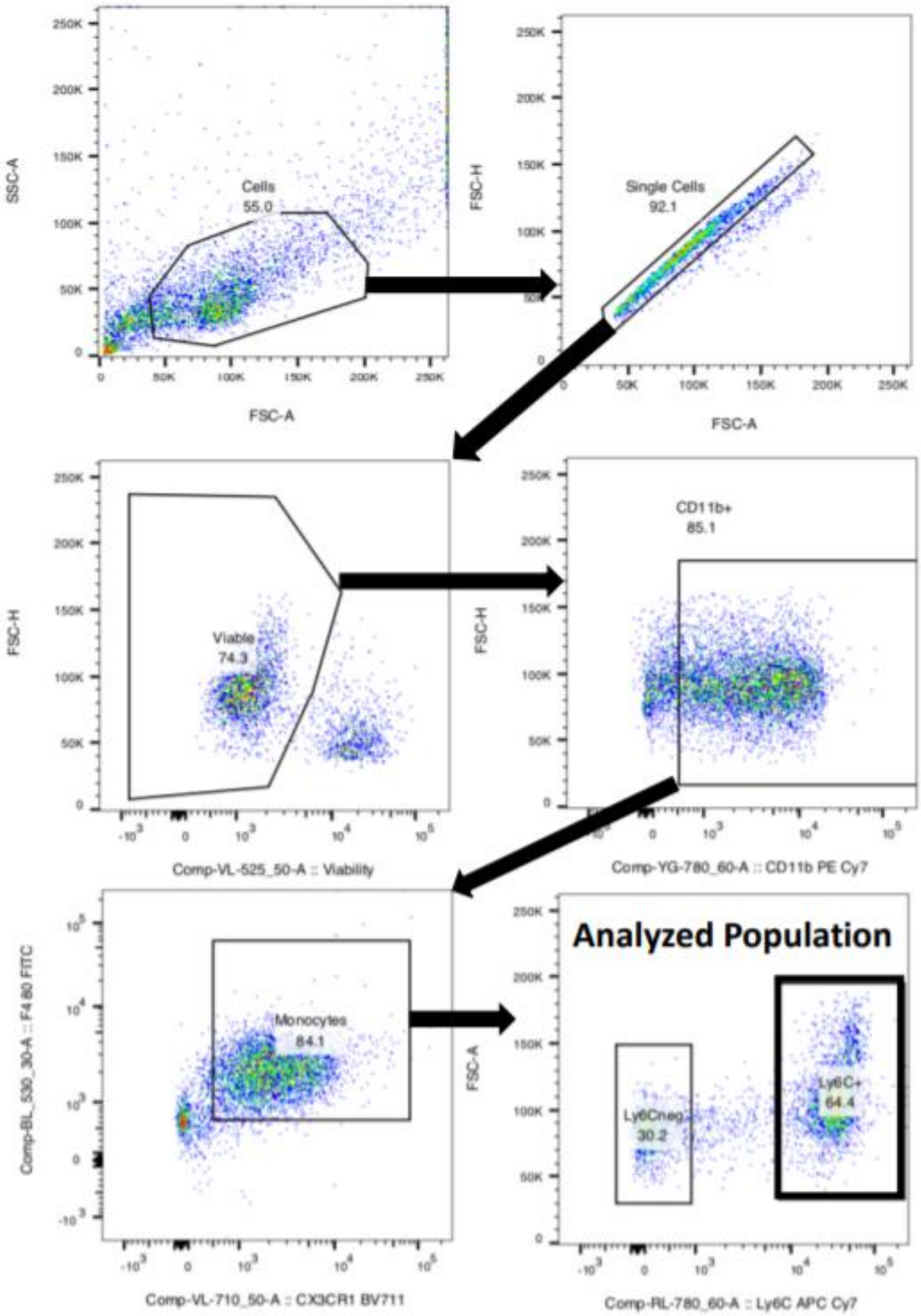

Figure S3. Flow cytometry gating strategy for monocytes.
